# An iNTT system for the large-scale screening of differentially expressed, nuclear-targeted proteins: cold-treatment-induced nucleoproteins in Rye (*Secale cereale* L.)

**DOI:** 10.1186/s12864-016-2548-y

**Published:** 2016-03-05

**Authors:** Xinyou Cao, Xueyan Chen, Yangna Liu, Zhaoshi Xu, Liancheng Li, Yongbin Zhou, Jianjun Liu, Zhendong Zhao, Ming Chen, Youzhi Ma

**Affiliations:** National Key Facility for Crop Genetic Resources and Genetic Improvement, Key Laboratory of Crop Genetics and Breeding, Ministry of Agriculture/Institute of Crop Sciences, Chinese Academy of Agricultural Sciences, Beijing, 100081 P.R. China; Crop Research Institute, Shandong Academy of Agricultural Sciences/National Engineering Laboratory for Wheat and Maize/Key Laboratory of Wheat Biology and Genetic Improvement in North Yellow and Huai River Valley, Ministry of Agriculture, 250100 Jinan, China; College of Agronomy, Northwest A&F University, Yangling, 712100 P.R. China

**Keywords:** Nuclear-targeted protein, iNTT system, After suppression subtraction method, Low-temperature stress, Rye

## Abstract

**Background:**

Nuclear proteins play critical roles in regulating mRNA transcription and processing, DNA replication, and epigenetic genome modification. Therefore, the ability to monitor changes in nuclear proteins is helpful not only to identify important regulatory proteins but also to study the mechanisms of actions of nuclear proteins. However, no effective methods have been developed yet. Rye is strongly resistant to various biotic and abiotic stresses; however, few genes have been functionally characterized to date due to the complexity of its genome and a lack of genomic sequence information.

**Results:**

We developed an integrative Nuclear Transportation Trap (iNTT) system that includes an improved nuclear transportation trap and utilizes the “after suppression subtraction” method. Oligonucleotides encoding a nuclear localization signal (NLS) or a transcription factor, GmAREB, were inserted into pLexAD or pLexAD-NES, respectively, and then transformed into yeast cells (EGY48). We showed that the pLexAD vector expressing a cDNA library in the iNTT system was more efficient for screening than the vector pLexAD-NES, which has previously been used in an NTT system. We used the iNTT system to screen a cDNA library of cold-treated rye. A total of 241 unique genes were identified, including 169 differentially expressed proteins; of these, 106 were of known and 63 were of unknown function. Moreover, 82 genes (49 %) among the 169 differentially expressed genes were predicted to contain an NLS domain. Thirty-three (31 %) of the 106 functionally known proteins have DNA-binding activity. To test the specificity of the nuclear proteins identified using the iNTT screen, four of the proteins differentially expressed in response to temperature stress, ScT1 (a heat shock protein), ScT36 (a MYB-like transcription factor), ScT133 (an ERF-like transcription factor) and ScT196 (a protein of unknown function), were studied in more depth. These proteins were shown to exclusively localize to the nucleus, and their expression levels were increased in response to low-temperature stress. To identify the function of these screened nuclear proteins, *ScT1-* and *ScT36-*transgenic *Arabidopsis* plants were constructed, and *ScT1* or *ScT36* overexpression was found to enhance tolerance to high-temperature or freezing stresses, respectively.

**Conclusions:**

The newly developed iNTT system provides an effective method for identifying nuclear-targeted proteins and monitoring induced expression levels. *ScT1* and *ScT36* might be good candidate genes for improving the stress tolerance of plants by genetic transformation.

**Electronic supplementary material:**

The online version of this article (doi:10.1186/s12864-016-2548-y) contains supplementary material, which is available to authorized users.

## Background

Nuclear proteins play critical roles in regulating mRNA transcription and processing, DNA replication, and epigenetic genome modification. Nuclear proteins generally contain a nuclear localization signal (NLS) sequence [1–3], a short peptide that mediates the transport of nuclear proteins into the nucleus. The NLS can be recognized by Kap or the importin α/β heterodimer. In yeast, approximately 27 % of proteins are targeted to the nucleus [4]. However, knowledge concerning nuclear proteins in plants is limited due to the lack of highly efficient screening methods and gene functional annotation information. Therefore, efficient screening methods are required for plant research.

Traditionally, the isolation and identification of nuclear proteins is achieved using conventional proteomic strategies or cell-based approached that tag proteins with an epitope or green fluorescent protein (GFP) for the detection of intracellular localization [5, 6]. However, these methods achieve low screening efficiency and are time-consuming. In 1998, the nuclear transportation trap (NTT) developed by Ueki et al. was used to screen for nuclear-targeted proteins in a human fetal brain cDNA library [7]. Rice cDNA libraries from three developmental stages have also been screened using the NTT system [8]. The main advantages of the NTT system for large-scale nuclear protein isolation are its efficiencies in cost, time, and labor. Although the NTT system achieves significantly greater screening efficiency than traditional methods, the efficiency of this technique, particularly in screening nuclear-targeted proteins, can be improved further. Moreover, the NTT system should be further improved to enable the screening of differentially expressed nuclear proteins in combination with other molecular biological approaches.

Rye (*Secale cereale* L.) is an important crop that exhibits strong resistance to various biotic and abiotic stresses [9]. The functional analysis of rye has lagged behind that of other cereals, possibly due the large size of its genome (~8 Gb) and the lack of available genomic information. The construction of genetic, physical, and QTL maps for rye has revealed the presence of genes (including *Lr26*, *Pm8*) that are related to its strong resistance to rust and mildew and to its aluminum tolerance [10]. However, few resistance genes have been identified in rye thus far; such genes would be important for the molecular breeding of cereals, particularly wheat. Wheat-rye translocations are widely used in wheat breeding to confer resistance against abiotic and biotic stresses, and various forms of the short arm of rye chromosome 1 (1RS) (e.g., 1AL.1RS, 1BL.1RS, and 1DL.1RS) have been introduced to confer disease and pest resistance to wheat (*Triticum aestivum*) [11, 12]; approximately 50 % of wheat varieties maintained by the International Maize and Wheat Improvement Center (CIMMYT) contain the 1BL.1RS translocation [13]. Therefore, the screening and functional analysis of resistance genes in rye is useful for improving wheat resistance via genetic transformation.

In this study, an integrative nuclear transportation trap (iNTT) was developed by integrating an improved NTT system and the “after suppression subtraction” method. In the iNTT system, the pLexAD vector was used to screen a cDNA library instead of the pLexAD-NES vector, which was previously used in NTT systems. The iNTT system demonstrated a higher screening efficiency than previous NTT systems when screening for NLS and GmAREB nuclear proteins. To identify temperature-resistant and temperature-responsive genes in rye, a low temperature-treated rye cDNA library was screened using the iNTT system; consequently, 241 unique genes were identified. Four nuclear proteins (ScT1, ScT36, ScT133, and ScT196) were selected for further functional analysis. Subcellular localization analysis indicated that all of the four candidate proteins were localized in the nucleus, and Q-RT-PCR analysis demonstrated that all four genes were expressed at higher levels in response to low-temperature stress. *ScT1* and *ScT36* overexpression can enhance high-temperature and freezing-stress tolerances in transgenic plants, respectively. In short, the iNTT system proved useful for isolating important nuclear-targeted proteins that are induced under various treatment conditions.

## Results

### Construction and screening efficiency of the iNTT system

**Fig. 1:**
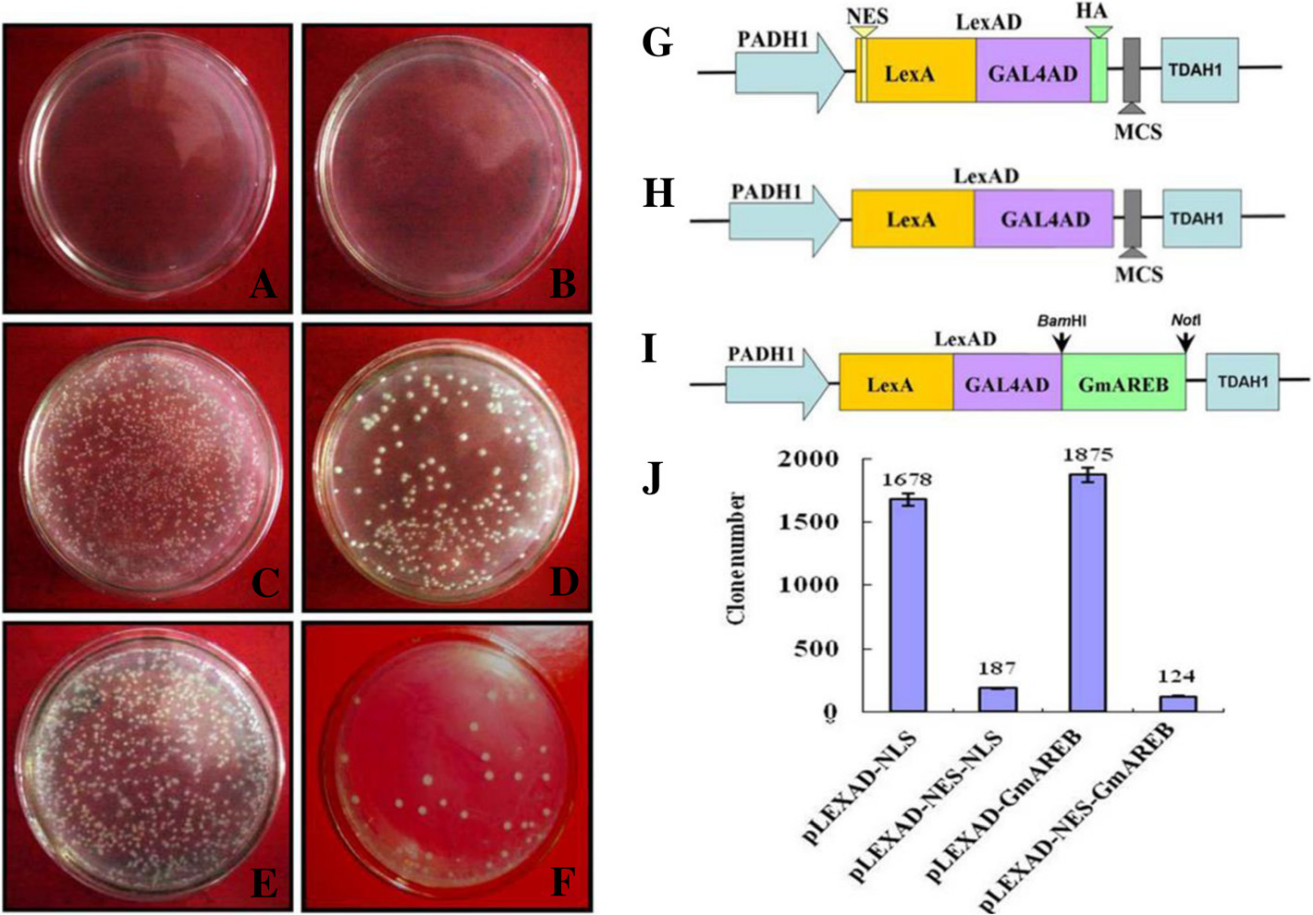
Vectors and screening efficiency of the iNTT system. **a** Yeast cells transformed with pLexAD. **b** Yeast cells transformed with pLexAD-NES. **c** Yeast cells transformed with pLexAD-NLS. **d** Yeast cells transformed with pLexAD-NES-NLS. **e** Yeast cells transformed with pLexAD-GmAREB. **f** Yeast cells transformed with pLexAD-NES-GmAREB. **g** The expression cassettes used in the NTT system. PADH1: yeast *alcohol dehydrogenase 1* (*ADH1*) gene promoter; LexA: DNA-binding domain of the LexA protein; GAL4AD: transactivation domain of the GAL4 protein; TDAH1: terminator of the *ADH1* gene. **h** The expression cassettes used in the iNTT system. **i** The expression cassettes used in the iNTT system fused to *GmAREB*. **j** The number of yeast clones transformed with the four vectors pLexAD-NLS, pLexAD-NES-NLS, pLexAD-GmAREB, and pLexAD-NES-GmAREB

In our iNTT system, the vector pLexAD contains the fragment LexAD, which comprises the DNA-binding domain of the LexA transcription factor and a transactivation GAL4 transcription factor domain (Fig. 1h). In a previously used NTT system, the vector pLexAD-NES was constructed using the fragment NES-LexAD, which encodes the nuclear export signal (NES) of the human immunodeficiency virus\ regulator of virion protein expression (HIV Rev) (Fig. 1g) [7, 8, 14].

To compare the screening efficiencies of these vectors, two differently sized proteins were evaluated: a 17 amino acid NLS from the SV40 large T antigen protein and a 438 amino acid bZIP-like transcription factor (*GmAREB*) [15]. A gene encoding the SV40 large T antigen NLS was inserted into pLexAD and pLexAD-NES to generate the fused vectors pLexAD-NLS and pLexAD-NES-NLS, respectively. Equal amounts (5 mg) of pLexAD, pLexAD-NES, pLexAD-NLS and pLexAD-NES-NLS DNA were transformed into yeast cells (EGY48), and the transformants were grown on SD medium lacking leucine and histidine (Leu-/His-) at 30 °C for 4 days. As shown in Fig. 1, pLexAD and pLexAD-NES transformants did not grow on selection medium (Leu-/His-) (Fig. 1a and b, respectively), unlike the pLexAD-NLS and pLexAD-NES-NLS transformants, which did grow (Fig. 1c and d, respectively). The number of pLexAD-NLS transformants was 9-fold higher than that of pLexAD-NES-NLS (*P* < 0.01; Fig. 1j) in three replicate experiments, suggesting that pLexAD can provide greater screening efficiency than pLexAD-NES.

To further evaluate the screening efficiencies of these vectors at detecting larger transcription factors, *GmAREB*, a bZIP-like transcription factor previously reported by our laboratory [15], was inserted into both pLexAD and pLexAD-NES to produce pLexAD-GmAREB and pLexAD-NES-GmAREB, respectively (Fig. 1i). Similar to the results obtained using NLS, yeast cells transformed with pLexAD-GmAREB or pLexAD-NES-GmAREB were able to grow on the selection medium (Fig. 1e and f, respectively), and the number of pLexAD-GmAREB transformants was 15-fold higher than that of pLexAD-NES-GmAREB (*P* < 0.01; Fig. 1j). Collectively, these results demonstrate that using the vector pLexAD and our iNTT system is more efficient for screening transcription factor genes than using the vector pLexAD-NES and the previously reported NTT system.

### Identification of cold-responsive nuclear proteins in rye using the iNTT system

Two rye cDNA libraries (control and 5 h cold treatment) were inserted into the vector pLexAD and transformed into the yeast strain EGY48. Nuclear proteins that were differentially expressed in response to cold stress were further identified using the “after suppression subtraction” method. A total of 312 and 517 positive clones with sequence lengths greater than 500 bp were selected and further sequenced in the control and cold-treated cDNA libraries, respectively. A total of 461 EST sequences were obtained from the two cDNA libraries (combined or overlapping between the two libraries). A BLAST analysis (based on e-values of less than 10^−5^ and identities > 90 %) of the 461 EST sequences further identified 241 unique genes that encoded high-confidence proteins. Among these 241 unique sequences, 72 were expressed in both libraries (the sequence identities of the 72 genes from the two cDNA libraries were greater than 98 %). Finally, 169 genes were considered to be cold-induced according to the “after suppression subtraction” method (Additional file 1: Table S1 and Table S2). Based on NCBI entries and annotations, these 169 unique proteins include 106 of known function and 63 of unknown function.

**Fig. 2:**
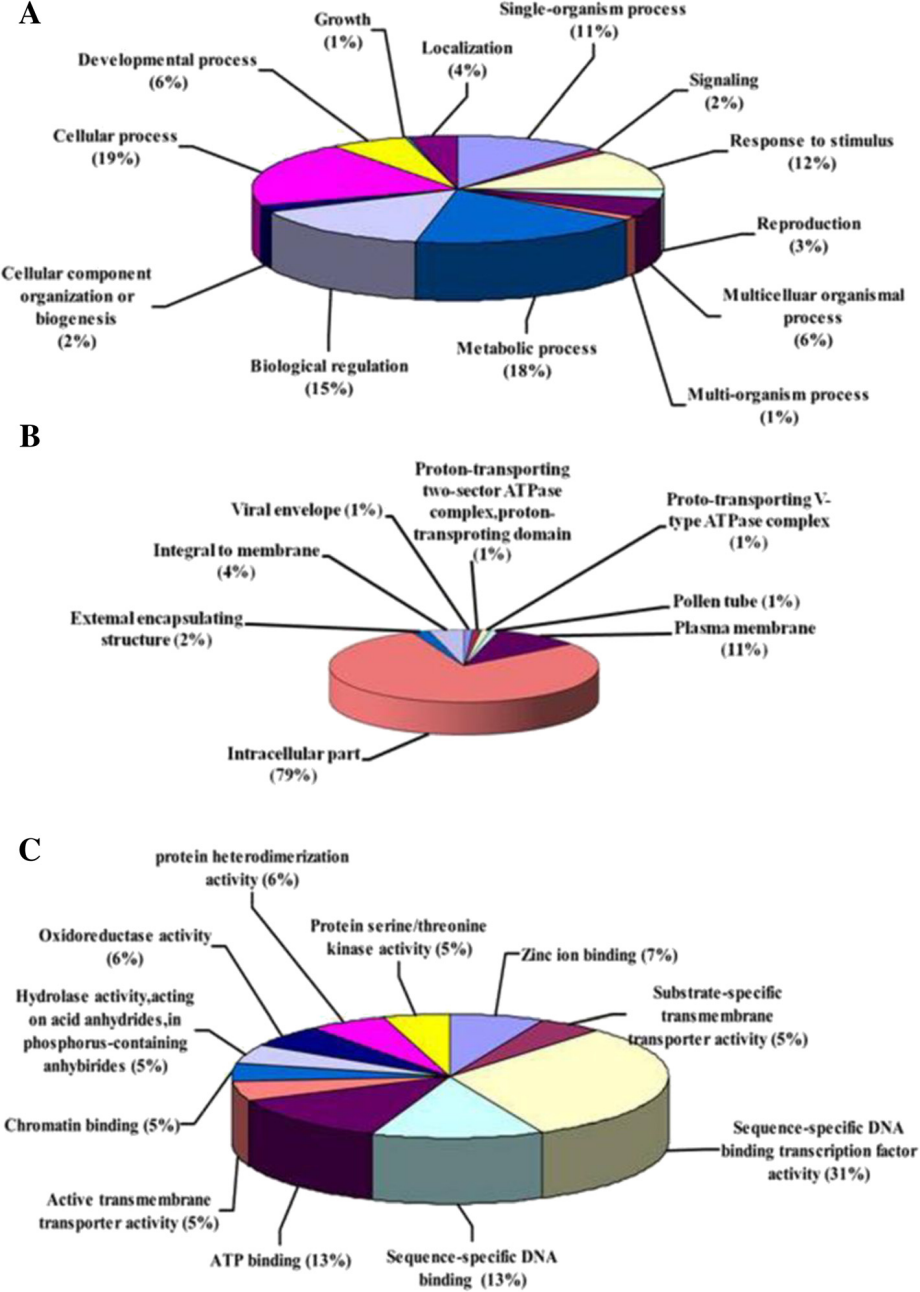
GO enrichment analysis of differentially expressed genes. Gene function classification of 169 genes that were differentially expressed between two rye cDNA libraries based on the biological process GO consortium (2 -**A**, 2nd level), the cellular component GO consortium (2 -**B**, 5th level) and the molecular function GO consortium (2-**C**, 5th level)

Using the GO classification scheme, these 106 known proteins were further categorized according to biological process (Fig. 2a), cellular component (Fig. 2b) and molecular function (Fig. 2c). The largest percentage of genes was involved in cellular processes when sorted by biological process (19 %, Fig. 2a). Of the genes sorted by cellular components, 79 % were intracellular (Fig. 2b), and of the genes sorted by molecular function, 31 % exhibited sequence-specific DNA-binding transcription factor activity (Fig. 2c). A PSORT analysis (http://psort.hgc.jp/form.html) suggested that 82 out of the 169 unique genes (49 %) function as transcriptional regulators, transcription factors and/or DNA/RNA-binding proteins. Importantly, these 82 nuclear proteins were only expressed in the 5-h cold-treated cDNA library and included ScT1 (heat shock protein, GenBank accession no. JQ685506), ScT36 (MYB-like transcription factor, GenBank accession no. KR584664), ScT133 (ERF-like transcription factor), and ScT196 (a protein of unknown function).

### Subcellular localization analysis of four cold-induced nuclear proteins from rye

**Fig. 3:**
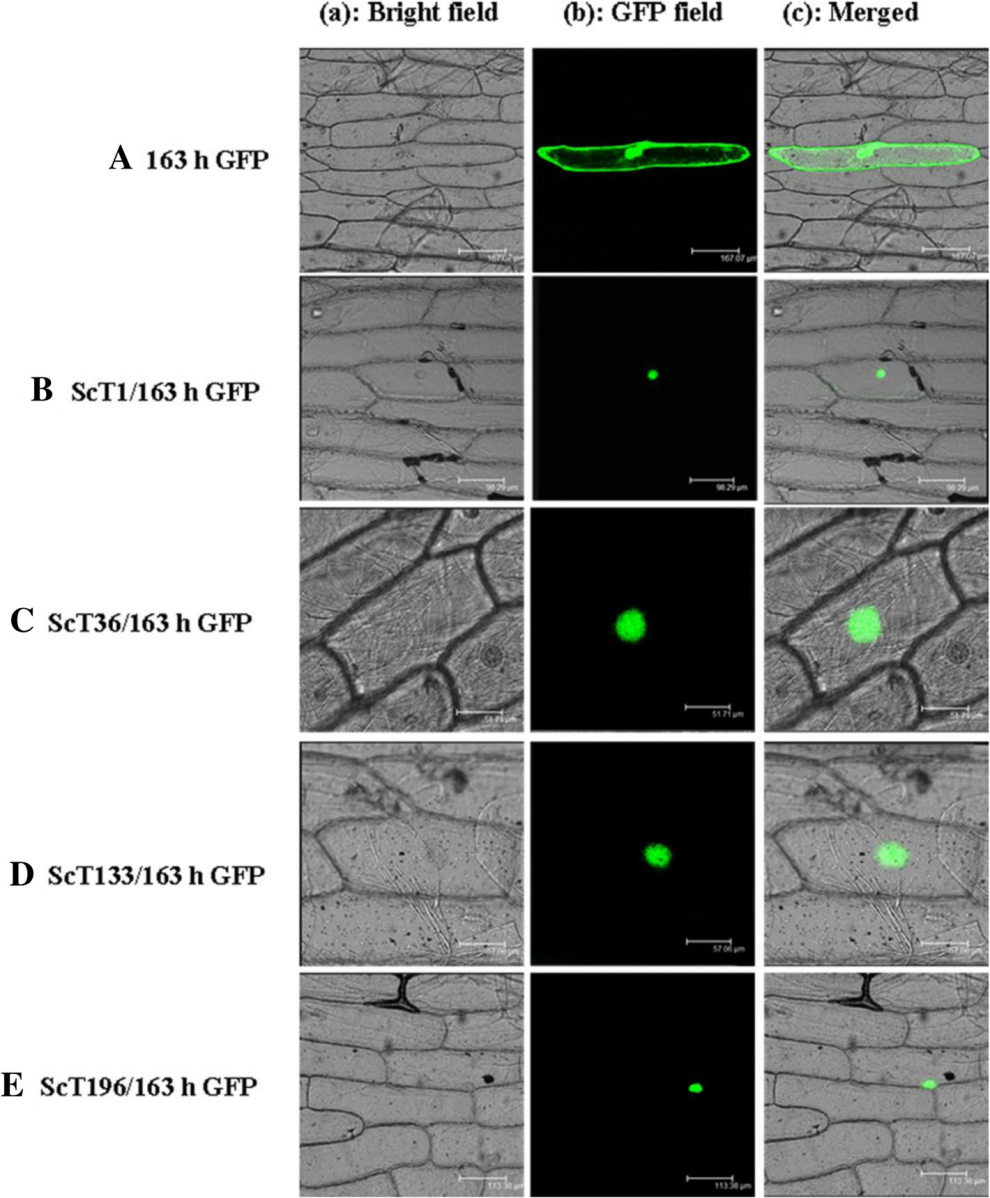
Nuclear localization of four rye proteins. **a** The control vector 35S::GFP, **b** 35S::ScT1/163hGFP, **c** 35S::ScT36/163hGFP, **d** 35S::ScT133/163hGFP and **e** 35S::ScT196/163hGFP were transiently expressed in onion cells. Column (*a*), merged images; column (*b*), GFP fluorescence; column (*c*), overlaid images. The images were visualized using a confocal microscope. The positive-control plasmid 35S::163hGFP encoded only GFP

To identify genes that are importantly responsible for cold tolerance in rye, four putative nuclear proteins, the expression levels of which were more than 10-fold higher in the 5-h cold treatment cDNA library (ScT1, ScT36, ScT133 and ScT196; Additional file 1: Table S1), were selected for further genetic and functional analyses. To study the subcellular localization of these four proteins, the corresponding full-length cDNA sequences were fused to the 5´ end of the 163hGFP gene under the control of the CaMV35S promoter, and the four resulting recombinant plasmids were transformed into onion epidermal cells for use in transient expression assays. As shown in Fig. 3, fluorescence was detected throughout the entire cell in transformants containing the positive control plasmid constructed using 163hGFP (Fig. 3a); in contrast, in cells transformed with ScT1, ScT36, ScT133 or ScT196, fluorescence was only detected in the nucleus (Fig. 3b, c, d, and e, respectively), suggesting that these four proteins were localized to the nucleus.

### Cold-induced expression patterns of four nuclear protein genes in rye

**Fig. 4:**
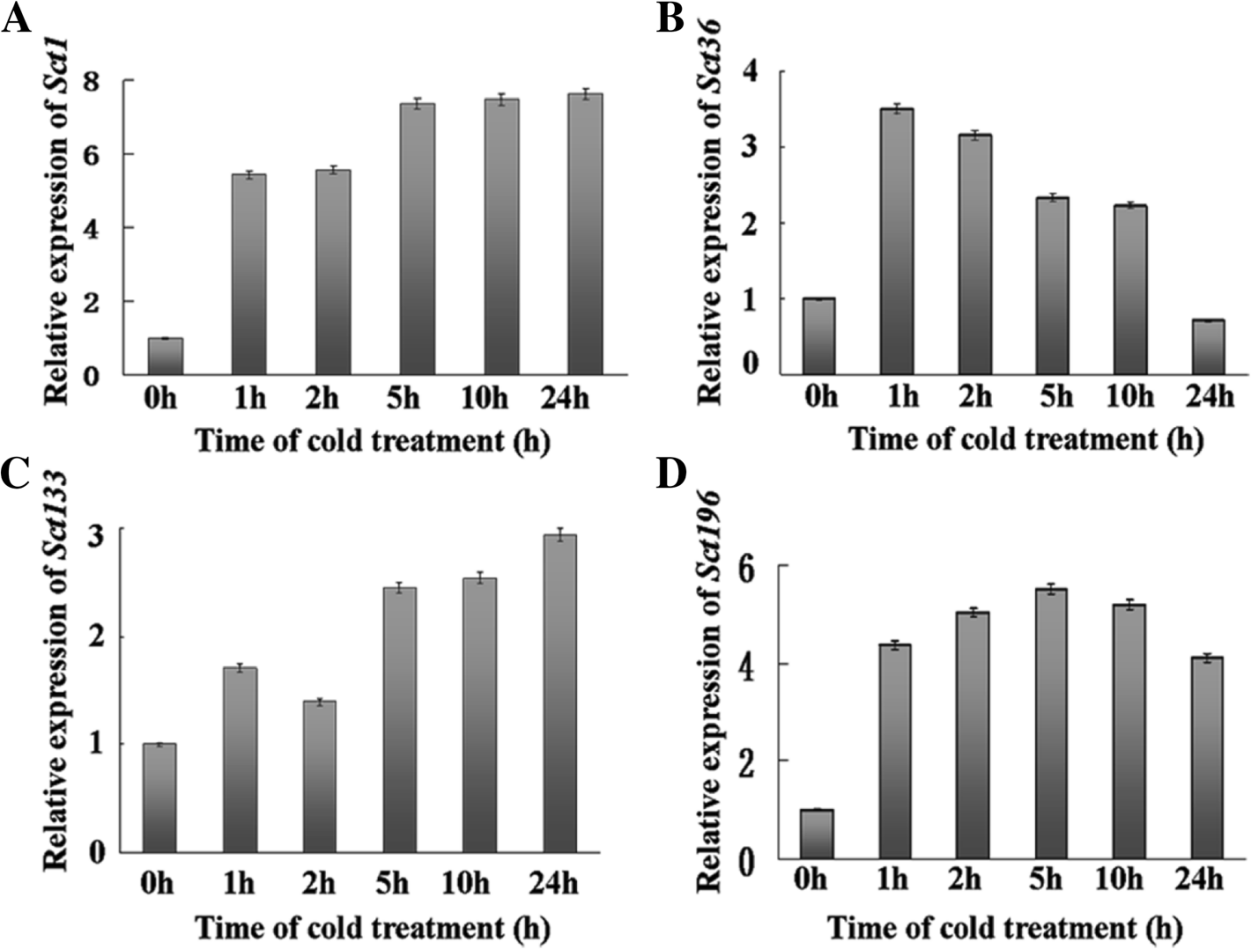
Expression profile analysis of four nuclear protein genes in rye. Total RNA was isolated from rye seedlings that were exposed to low temperatures for various times. Total RNA (2 μg) was reverse-transcribed into first-strand cDNA for qRT-PCR. The *actin* gene was amplified as a control. The expression levels are presented as values relative to the average expression of the *actin* gene. **a** Expression of the *ScT1* gene. **b** Expression of the *ScT36* gene. **c** Expression of the *ScT133* gene. **d** Expression of the *ScT196* gene. The mean and SE of three biological and technical replicates are presented

The expression patterns of the four nuclear protein genes after low-temperature treatment were analyzed using Q-RT-PCR. As shown in Fig. 4, the transcripts of the four nuclear protein genes were highly upregulated after cold treatment. The increased expression of *ScT1* and *ScT133* was time-dependent and reached relatively high levels after 24 h of cold treatment (Fig. 4a and c, respectively). The expressions of *ScT36* and *ScT196* exhibited a biphasic pattern with acute increases within the first 1 h and 5 h of cold treatment, respectively, followed by decreases (Fig. 4b and d, respectively). These results suggest that the expression of these four genes is induced by and is responsive to cold stress and that the iNTT system can be reliably used to screen for differentially expressed nuclear protein genes in a cDNA library.

### Overexpression of *ScT36* increased tolerance to low-temperature stresses in transgenic plants

**Fig. 5:**
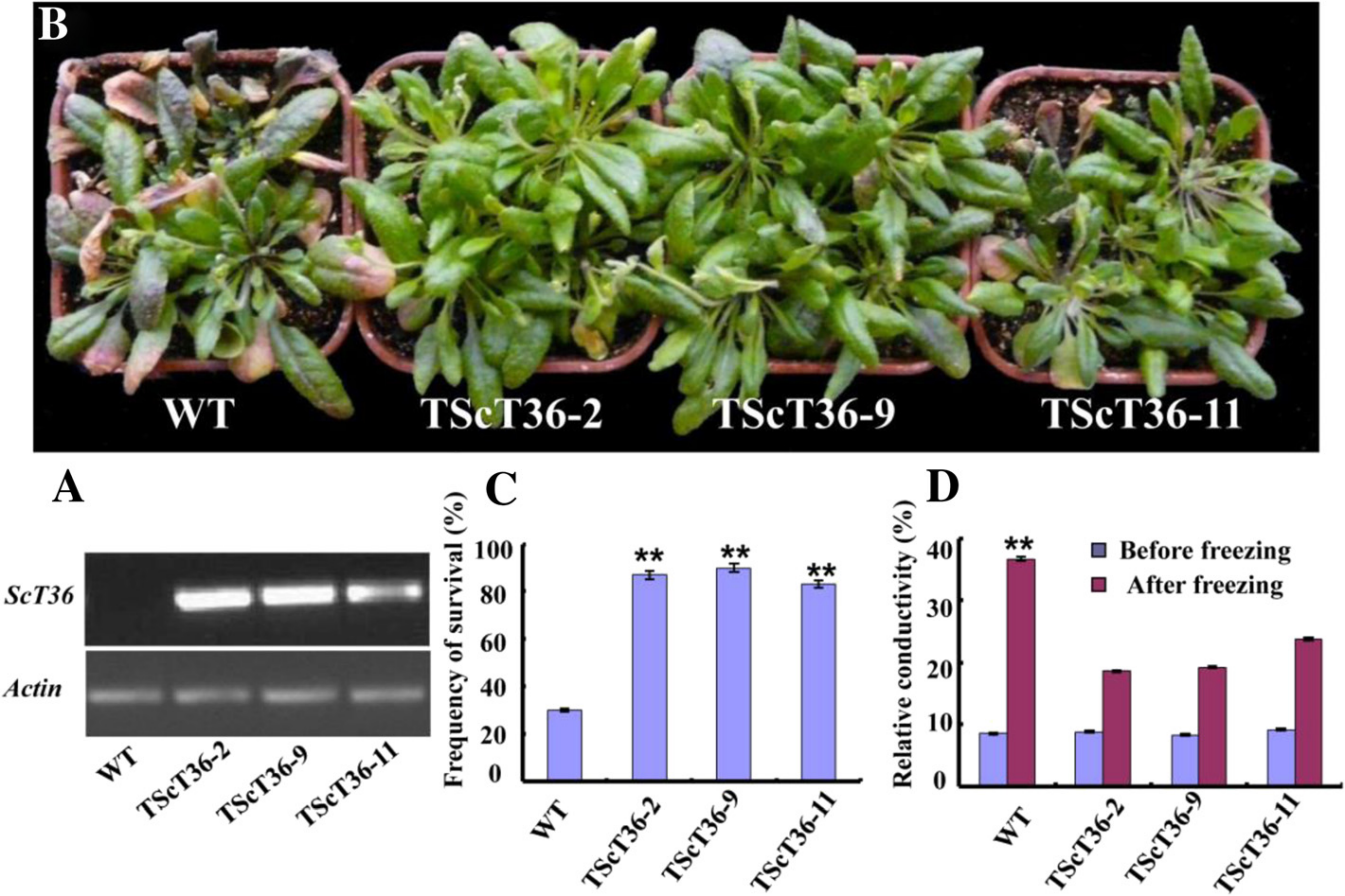
Freezing-stress tolerance and relative electrical conductivity of *ScT3-*transgenic *Arabidopsis* plants. **a** RT-PCR analysis of T3 transgenic plants. **b** Growth of WT (*left*) and transgenic (*right*) lines after freezing treatment. The plants were placed in a chamber at −10 °C for 2 h and then moved to normal conditions. **c** Survival frequency was determined after the plants recovered for 7 days under normal conditions. ** indicates significantly different values between WT and transgenic plants (*P* < 0.01). **d** Relative electrical conductivity of WT and transgenic *Arabidopsis* were determined after exposure to −10 °C for 2 h. ** indicates significantly different values before and after the treatment (*P* < 0.01). The results shown represent the means of three replicates ± SD

RT-PCR detection confirmed that *ScT36* was transcribed in T3 transgenic *Arabidopsis* lines (Fig. 5a). Three independent T3 transgenic *Arabidopsis* lines overexpressing *ScT36* were subjected to freezing analyses. After low-temperature stress (−10 °C) for 2 h, wild-type (WT) *Arabidopsis* leaves wilted, turned purple and died; in contrast, the leaves of *ScT36-*transgenic plants became yellow after treatment but returned to green after 7 days of recovery time (Fig. 5b). The leaf survival rates of the three independent transgenic lines TScT36-2, TScT36-9, and TScT36-11 were 87, 90 and 83 %, respectively, whereas that of WT was only 30 % (*P* < 0.01; Fig. 5c).

Relative electrical conductivity can be used as a measure of cell membrane stability, and to further understand the role of *ScT36* in the cold stress response, the relative conductivities of the transgenic *Arabidopsis* lines were measured before and after freezing. Before freezing, no differences in relative conductivity were apparent between the WT and transgenic plants (Fig. 5d). After two hours of freezing treatment (−10 °C), the relative conductivity values of all of the plants were increased; however, the conductivity of the three transgenic lines TScT36-2, TScT36-9, and TScT36-11 were 18.6, 19.2 and 23.8 %, respectively, which was lower than that of the control (36.7 %) (*P* < 0.01; Fig. 5d). These results suggest that *ScT36* overexpression decreases chilling injury and enhances the tolerance of transgenic plants to freezing conditions. Similar freezing analyses were also performed on an *ScT1-*transgenic plant; however, no difference was observed between the transgenic and WT plants under low-temperature stress.

### Overexpression of *ScT1* increased tolerance to high-temperature stresses in transgenic plants

**Fig. 6:**
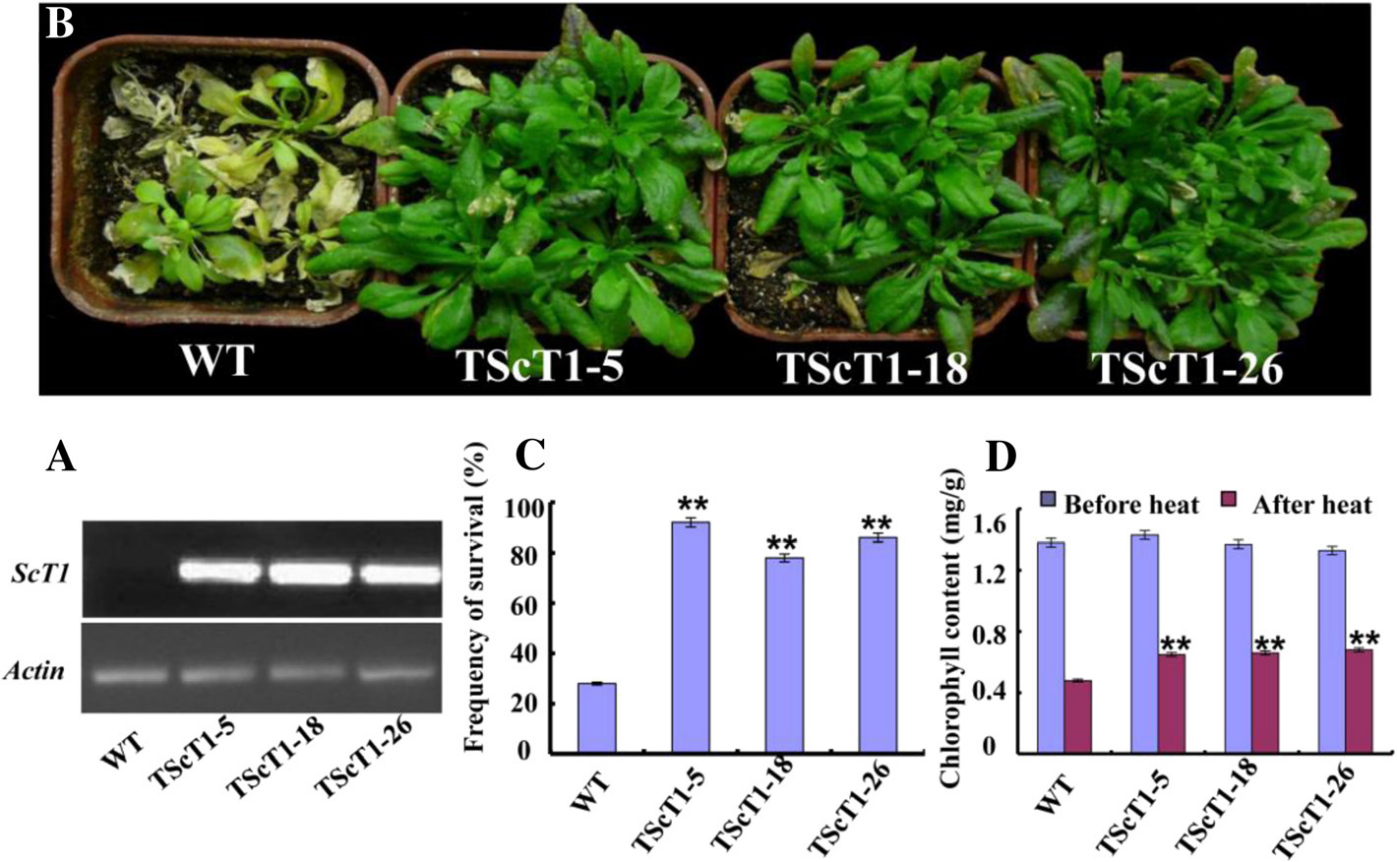
Heat-stress tolerance and chlorophyll levels in *ScT1*-transgenic *Arabidopsis* plants. **a** RT-PCR analysis of T3 transgenic plants. **b** Growth of WT (*left*) and transgenic (*right*) lines after heat treatment. Plants were exposed to 42 °C for 3 h and then placed in normal conditions for 7 days. **c** Survival frequency was determined after the plants recovered for 7 days under normal conditions. The results shown represent the averages of three replicates ± SD. ** indicates significantly different values between WT and transgenic plants (*P* < 0.01). **d** The chlorophyll levels (in mg/g fresh weight) in leaves were measured after heat treatment for 3 days. ** indicates significantly different values before and after treatment (*P* < 0.01). The results shown represent the means of three replicates ± SD

RT-PCR detection confirmed that *ScT1* was transcribed in T3 transgenic *Arabidopsis* lines (Fig. 6a). To investigate whether *ScT1* plays a role in response to heat stress, we heat-treated *ScT1*-transgenic plant at 42 °C for 3 h. After 7 days of recovery time, WT plants began to exhibit visual symptoms of heat-induced damage, such as leaf yellowing and severe wilting (Fig. 6b); in contrast, the transgenic plants exhibited stronger tolerance to the heat treatment, and there was no obvious effect on transgenic plant development and growth. The leaf survival rates of the three independent transgenic lines TScT1-5, TScT1-18, and TScT1-26 were 92, 78, and 86 %, respectively, whereas that of WT was only 28 % (*P* <0.01; Fig. 6c). The chlorophyll levels in the transgenic and WT plants, an important indicator of photosynthesis [16], were measured after heat treatment for 3 h. As shown in Fig. 6d, the chlorophyll levels of the three independent transgenic lines TScT1-5, TScT1-18, and TScT1-26 were 1.43 mg/g, 1.37 mg/g and 1.33 mg/g before heat treatment and were not significantly different from that of WT plants (1.38 mg/g). Although heat treatment significantly decreased the chlorophyll contents in all of the plants, the remaining chlorophyll level was higher in transgenic than in WT plants. The chlorophyll levels of the three independent transgenic lines TScT1-5, TScT1-18, and TScT1-26 were 0.65 mg/g, 0.66 mg/g, and 0.68 mg/g, respectively, whereas that of WT was only 0.48 mg/g (*P* < 0.01; Fig. 6d). These data suggest that *ScT1* overexpression can improve heat stress tolerance in transgenic plants.

## Discussion

The screening efficiency of the iNTT system established in this study was higher than that obtained using the previous NTT system. Upon transforming the same amount of DNA encoding a short peptide *NLS* or the transcription factor *GmAREB* into yeast, the resulting numbers of transformants using pLexAD and our iNTT system were 9- and 15-fold higher, respectively, compared to pLexAD-NES and the NTT system (Fig. 1). The pLexAD-NES vector is commonly used for screening with the NTT system. Ueki reported that yeast cells transformed with pLexAD-NES could not grow on selection medium, whereas yeast cells transformed with pLexAD grew well on selection medium [7]. Ueki et al. suggested that LexAD fusion proteins could enter the nucleus by passive diffusion and that the NES sequence probably prevented the generation of false-positive clones [7]. The incorporation of an NLS into pNES-LexAD was sufficient for colony formation, and although the NES-NLS-LexAD fusion protein might shuttle continuously between the cytoplasm and the nucleus, the retention period of the chimera in the nucleus was sufficient for reporter gene transactivation. Mouse embryo and rice cDNA libraries were employed for further applications of the NTT system to isolate nuclear proteins [8, 17]. However, in our study, we found that yeast cells transformed with pLexAD did not grow on selection medium (Fig. 1a), and the same result has been reported in numerous repeated tests [18–20]. Therefore, we speculate that either the passive diffusion of LexAD described by Ueki et al. did not occur or the passive diffusion of the LexAD protein was insufficient to enable nuclear entry [7]. Moreover, no NLS sequence is present in the fusion protein encoded by the LexA DNA-binding domain and the GAL4 transactivation domain [21]. Therefore, a redundant NES sequence would not only fail to reduce false positives but would also reduce the screening efficiency of the NTT system, possibly explaining the difference in screening efficiencies observed between the iNTT and the NTT systems. Thus, unlike transcriptomic sequencing analyses or proteomic approaches, the NTT system saves cost, time, and labor, and the screening efficiency of the iNTT system was further improved for large-scale nuclear protein screening compared with the previous NTT system.

The iNTT system can be applied to screens for novel nuclear proteins and to study the dynamic composition of nucleoproteins under biotic and abiotic stresses, and these types of analyses are important to study gene expression regulatory mechanisms and to isolate key regulatory genes after treatment. Winter rye is the most frost-resistant cereal [9], and screening for cold tolerance-related genes in rye would be useful for improving the cold tolerance of other cereals such as wheat or rice. In this research, after screening two rye libraries obtained using control and 5 h low-temperature treatments, shared sequences from both libraries were eliminated using the “after suppression subtraction” method, resulting in 241 unique sequences; of these sequences, 169 were differentially expressed after treatment (Additional file 1: Table S1 and Table S2). Of these differentially expressed genes, 31 % were annotated as having DNA-binding transcription factor activity according to GO classification (Fig. 2c). Moreover, half of the proteins of known function (49 %) were predicted to localize to the nucleus (Additional file 1: Table S1 and Table S2), and four proteins (ScT1, ScT36, ScT133, and ScT196) were identified as nuclear-targeted and highly expressed upon cold treatment (Figs. 3 and 4). Moreover, the iNTT system was successfully applied to screen soybean and wheat libraries in our laboratory, and many nuclear proteins, such as *GmNAC2a* and *GmRZFP1*, were shown to be involved in abiotic or biotic stress response pathways that have been previously published in Chinese [18–20]. These results indicate that the iNTT system is an effective and accurate method that can be used to screen for nuclear-targeted proteins and to monitor treatment-induced protein expression. Currently, we are also developing another method combining NTT and suppression subtractive hybridization [22] for screening biotic and abiotic treatment-induced nucleoproteins.

To date, few genes have been functionally characterized in rye. In this study, we observed that the expression levels of four nuclear proteins were more than 10-fold higher after 5 h cold treatment, and they were selected for further analyses. *ScT36* overexpression in transgenic plants enhanced freezing tolerance (Fig. 5), whereas *ScT1* overexpression in transgenic plants enhanced extreme temperature tolerance (Figs. 5 and 6, respectively). *ScT1* is a member of the heat shock protein (Hsp) family, and Hsp-like genes have been isolated from several plant species and are widely involved in the response to various biological and abiotic stresses, including low and high temperatures, drought, high salinity, exposure to disease and pests, and SA and abscisic acid (ABA) treatments [23]. The overexpression of *ScT1* in transgenic plants enhanced heat-stress tolerance (Fig. 6) but not low-temperature tolerance (data not shown). Therefore, specific knowledge is required to further understand how Hsp-like proteins enter into the nucleus and affect plant tolerance of heat stress. MYB transcription factors comprise the largest transcription factor family in plants. Members of this family play key roles in plant development, secondary metabolism, hormone signal transduction, disease resistance and abiotic stress tolerance [24, 25]. *ScT36* (an MYB-like transcription factor) overexpression in transgenic *Arabidopsis* resulted in enhanced cold tolerance, consistent with previous studies, but not heat-stress tolerance (data not shown). For example, overexpressing *OsMYB3R2* led to stronger cold tolerance and an increased mitotic index in transgenic rice [26], and enhanced freezing-stress tolerance was observed in *Arabidopsis* overexpressing *OsMYB4* [27]. Transgenic *Arabidopsis* expressing *AtMYB15* exhibited hypersensitivity to exogenous ABA and improved drought and cold tolerance [28, 29]. These results show that *ScT1* and *ScT36* can play important roles in the heat and cold response responses of *Arabidopsis* and might be valuable for further research to improve tolerance to heat and cold stresses in other crops.

## Conclusions

In summary, the newly iNTT system was developed by integrating an improved NTT system and the “after suppression subtraction” method. The results suggest that iNTT system is an effective method for identifying nucleartargeted proteins and monitoring their induced expression levels, particularly for species for which genomic sequence information is currently unavailable. *ScT1* and *ScT36* overexpression can enhance high-temperature and freezing-stress tolerances in transgenic plants, respectively. *ScT1* and *ScT36* might be good candidate genes for improving the stress tolerance of plants by genetic transformation.

## Methods

### Plasmid construction

A LexA-GAL4AD (pLexAD) expression cassette was constructed by inserting a PCR-amplified GAL4AD domain encoding an pACT2 HA epitope tag (Clontech, Tokyo, Japan) into the *Eco*RI and *Bam*HI sites of pLexA (Clontech, Tokyo, Japan) (Fig. 1h). A NES-LexA-GAL4AD (pLexAD-NES) expression cassette was constructed by inserting a synthesized HIV-1 Rev NES sequence (positive sequence: 5´-ACTTCAGCTACCACCGCTTGAGAGACTTACTCTTGATTT-3´; reverse complementary sequence: 5´- AAATCAAGAGTAAGTCTCTCAAGCGGTGGTAGCTGAAGT-3´) (Life Technologies Corporation, Carlsbad, CA, USA) into the *Hpa*I site of pLexAD (Fig. 1g). NLS fusion plasmids containing either the NLS–LexAD fusion protein (pLexAD-NLS) or the NLS-NES–LexAD fusion protein (pLexAD-NES-NLS) were then constructed by inserting a synthesized SV40 large T antigen NLS sequence (positive sequence: 5´- GATCCTTAATTCCCGAGCCTCCAAAAAAGAAGAGAAAGGTCGAATTGGGTACCGCCC −3´; reverse complementary sequence: 5´- TCGAGGGCGGTACCCAATTCGACCTTTCTCTTCTTTTTTGGAGGCTCGGGAATTAAG −3´) (Life Technologies Corporation, Carlsbad, CA, USA) between the *Bam*HI and *Xho*I sites of pLexAD and pLexAD-NES, respectively. Similarly, GmAREB fusion plasmids containing either the GmAREB–LexAD fusion protein (pLexAD-GmAREB) or the GmAREB-NLS–LexAD fusion protein (pLexAD-NLS-GmAREB) were constructed by inserting a gene encoding the *Glycine max* ABA-responsive element-binding (*GmAREB*) transcription factor between the *Bam*HI and *Not*I sites of pLexAD and pLexAD-NES, respectively (Additional file 2: Figure S1).

### Rye cDNA library construction

Rye (*Secale cereale* L. var. AR132) seedlings were grown at 25 °C for 14 d and then subjected to 4 °C cold stress in a temperature-controlled chamber. In previous research, we observed that some cold-responsive genes were expressed at a higher level after 5 h of cold treatment than under normal conditions or at other time points (data not shown). Therefore, leaves were harvested before (baseline control) and after 5 h of cold treatment, immediately frozen in liquid nitrogen and stored at −80 °C for future RNA extraction. Total RNA was extracted using the RNAprep Pure Plant kit (Tiangen Biotech, Beijing, China). Poly(A) + RNA was prepared from the isolated total RNA using the Oligotex™-dT30 (Super) mRNA Purification Kit (Takara, Japan). cDNA libraries were then prepared using a cDNA Library Construction Kit (Takara, Japan) with the following modifications: 5 μg poly(A) + RNA was used to synthesize first-strand cDNA using the Oligo (dT)18 Anchor Primer and M-MLV reverse transcriptase, and second-strand synthesis was performed using the SuperScript® Double-Stranded cDNA Synthesis Kit (Invitrogen, Carlsbad, CA, USA). After treatment with T4 DNA polymerase to generate blunt ends, a double-stranded adaptor containing *Bam*HI/*Sma*I adaptor sticky ends was ligated to both ends of the cDNA using T4 DNA ligase. The double-stranded cDNA was then digested using *Not*I, and small cDNA fragments were removed using a spin column (Clontech, Tokyo, Japan). pLexAD-GmAREB was digested using *Bam*HI and *Not*I, and only the pLexAD fragment was recovered. The purified cDNA was ligated to pre-digested pLexAD plasmid DNA using T4 DNA ligase. The construct was then transformed into *E. coli* via electroporation, and the transformants were incubated on a LB/amp plate at 37 °C for 12 h. All clones were then incubated in liquid LB/amp medium at 37 °C for 12 h, and the plasmids were extracted and purified using the TIANprep Mini Plasmid Kit (Tiangen Biotech, Beijing).

### Screening of yeast cells and the sequencing of positive colonies

Yeast cells (EGY48, which were kindly provided by Prof. Rongfeng Huang, Institute of Biotechnology Research, Chinese Academy of Agricultural Sciences) were transformed with the pLexAD, pLexAD-NES, pLexAD-NLS, pLexAD-NES-NLS, pLexAD-GmAREB, and pLexAD-NES-GmAREB plasmids (5 mg DNA each) or with the constructed rye cDNA libraries according to the Yeast Protocols Handbook (Clontech, Tokyo, Japan). The transformants were grown on agar plates containing synthetic dropout (SD) medium lacking leucine and histidine (Leu^−^/His^−^) at 30 °C for 2 to 7 days to detect LEU2 reporter gene expression. This screening was repeated at least three times. Subsequently, all colonies were grown in liquid SD medium (Leu-/His-) at 30 °C for 18 h, and plasmids were then extracted using the TIANprep Yeast Plasmid DNA Kit (Tiangen Biotech, Beijing). Each cDNA insert was amplified from the purified plasmids by PCR with the primer combination of 5’-GCGTTTGGAATCACTACAGGGATGTTTAATACCA-3’ and 5’-GGGGAGCGATTTGCAGGCATTTGC-3’ and the following reaction conditions: 3 min denaturation at 94 °C; 35 cycles of 30 s at 94 °C, 35 s at 56 °C and 60 s at 72 °C; and a final extension at 72 °C for 10 min. The PCR products were resolved using 1.2 % agarose gels and visualized using a Gel Doc EQ System (Bio-Rad, Richmond, USA). Amplified fragments longer than 500 bp were retained and sequenced. Full-length cDNA sequences were identified between translation-initiating and stop codons, and proteins translated from cDNAs without stop codons were considered protein fragments. Homology searches were performed using GenBank (http://blast.ncbi.nlm.nih.gov/Blast.cgi) with the threshold (e-value cut-off) set at e10^−5^. The sequence identity of each protein was determined based on the best match in the BLAST query, and an identity > 90 % was considered a high-confidence match. Sequences shared by both libraries (identity > 98 % and having the same annotated amino acid sequence in NCBI) were eliminated; this was called “after suppression subtraction.” The NLSs of candidate proteins were searched using the PSORT program by accessing the PSORT Web server (http://psort.hgc.jp/form.html), and all of the sequences were imported into Blast2GO for GO analysis (www.blast2GO.com) [30].

### Subcellular localization analysis

Four candidate genes (*ScT1*, *ScT36*, *ScT133* and *ScT196*) were subjected to subcellular localization analysis. Full-length *ScT36* and *ScT196* were obtained from the rye cDNA library. To obtain the full-length sequences of *ScT1* and *ScT133*, rapid amplification of the 5´ and 3´ ends of the *ScT1* and *ScT133* sequences and RT-PCR were performed using a 5’-RACE kit (Takara, Japan) and an RT-PCR kit (Life Technologies Corporation, Carlsbad, CA, USA), respectively. The cDNA fragments containing the coding regions of *ScT1*, *ScT36*, *ScT133* and *ScT196* were fused to the N-terminus of the *163hGFP* gene under the control of CaMV35S. The GFP-fusion plasmids and the 163hGFP control plasmid were transformed into onion (*Allium cepa* L.) epidermal cells by particle bombardment. The transformed tissues were cultured on Murashige and Skoog (MS) medium at 25 °C for 16 h. Transient expression of GFP-fusion proteins was observed using a laser confocal scanning microscope (Leica Microsystems, Heidelberg, Germany) as previously described by Cao et al. [31].

### Expression analysis of four nuclear protein genes by quantitative RT-PCR

Rye (AR132) seedlings were grown at 25 °C for 14 d and then subjected to cold stress in a 4 °C chamber; the leaves were harvested after 0, 1, 2, 5, 12, and 24 h of treatment. Total RNA was extracted using TRIzol reagent according to the manufacturer’s protocol (Tiangen Biotech, Beijing). First-strand cDNA was synthesized from 2 μg of RNA per sample using an RNA PCR Kit (Takara, Japan). The transcriptional levels of four genes (*ScT1*, *ScT36*, *ScT133* and *ScT196*) were quantified using real time quantitative RT-PCR (Q-RT-PCR). Q-RT-PCR analyses were conducted using an ABI Prism 7000 system (Applied Biosystems, USA) according to the modified protocol of Livak and Schmittgen [32]. The *actin* gene, which is expressed constitutively in wheat [33], was used as an internal control (the gene-specific primers used are presented in Additional file 1: Table S3). Transcription analyses of the four genes were performed using gene-specific primers (Additional file 1: Table S3) and the following reaction conditions: 95 °C for 3 min, followed by 41 cycles of 95 °C for 30 s and 60 °C for 45 s. Non-templates were included as negative controls, and the Ct value of each target gene was normalized to that of the *actin* gene. The relative gene expression level was derived from 2^-△△CT^ [32]. The transcriptional analysis was performed at least three times to confirm the accuracy of the results. The data were analyzed using Microsoft Excel. The relative expression levels of the four genes were calculated as the fold-increase of the gene expression level over the baseline control (0 h).

### Generation of transgenic *Arabidopsis* plants

*A. thaliana* ecotype Columbia-0 (Col-0, abbreviated WT in this paper) was used as the wild type control for all of the experiments performed in this study. For phenotypic analysis, plants were cultivated in a growth room with long-day growth conditions (16 h white light, 80–100 μmol · m^−2^ · s^−1^/8 h dark) at 21 ± 2 °C unless otherwise indicated. To construct expression vectors for transformation, *ScT1* and *ScT36* were ligated into the vector pBI121 to be expressed by the CaMV35S promoter. *Arabidopsis* Col-0 plants at the flower-budding stage were transformed using the floral dip method under vacuum conditions previously described [34, 35]. Transgenic plants were identified by RT-PCR, and the RNA levels isolated from T3 transgenic plant leaves were normalized using actin as control. Total RNA was extracted using TRIzol reagent according to the manufacturer’s protocol (Tiangen Biotech, Beijing). First-strand cDNA was synthesized from 2 μg RNA per sample using an RNA PCR Kit (Takara, Japan). All of the RT-PCR experiments were performed at least three times, with three or more samples time. Transcriptional analysis of the two genes was performed using gene-specific primers (Additional file 1: Table S3), and the reaction conditions were as follows: 3 min denaturation at 94 °C; 27 cycles of 30 s at 94 °C, 40 s at 58 °C and 30 s at 72 °C; then a final extension at 72 °C for 10 min. The PCR products (20 μL) were resolved on 1.2 % agarose gels and visualized with the Gel Doc EQ System (Bio-Rad, Richmond, CA, USA). Then, positive transgenic plants were used for further physiological and biochemical analyses.

### Analysis of the heat and freezing tolerances of transgenic plants

Transgenic and WT *Arabidopsis* plants were grown for 5 weeks in a growth room and then subjected to different heat or freezing treatments. Fifty plants from each line were used for each stress treatment. For heat treatment, the plants were grown at 42 °C for 3 h and then placed under normal conditions for 7 days. The survival rate was determined, and of the plants was measured as described by Aono et al. [36]. For the freezing treatment, transgenic and WT *Arabidopsis* plants were exposed to −10 °C for 2 h and then returned to normal conditions for 7 days. The survival rate was determined, and the chlorophyll content and relative electrical conductivity of the plants was measured as described by Hu et al. [37].

### Availability of supporting data

The data sets supporting the results of this article are included within the article and its additional files.

## Additional files

Additional file 1: Table S1. Related Unigenes using nuclear transportation trap (NTT) system and “after suppression subtractive” method. **Table S2.** Remove Unigenes using “after suppression subtractive” method from two libraries. **Table S3.** PCR primers for Q-RT-PCR analysis of gene expression. (DOC 327 kb) Additional file 2: Figure S1. Experimental protocol of iNTT system method. (DOCX 538 kb)

## Abbreviations

GmAREB: Glycine max ABA-responsive element-binding
iNTT: integrative Nuclear Transportation Trap
NES: nuclear export signal
NLS: nuclear location signal
NTT: nuclear transportation trap
pLexAD: A LexA-GAL4AD
pLexAD-NES: A NES-LexA-GAL4AD
