# Supplementary material for: An iNTT system for the large-scale screening of differentially expressed, nuclear-targeted proteins: cold-treatment-induced nucleoproteins in Rye (Secale cereale L.)
Source: BMC Genomics. 2016 Mar 5;17:189. doi: 10.1186/s12864-016-2548-y (PMC4779243; doi:10.1186/s12864-016-2548-y)
Supplement: Additional file 1: Table S1. — Related Unigenes using nuclear transportation trap (NTT) system and “after suppression subtractive” method. Table S2. Remove Unigenes using “after suppression subtractive” method from two libraries. Table S3. PCR primers for Q-RT-PCR analysis of gene expression. (DOC 327 kb) [file 12864_2016_2548_MOESM1_ESM.doc]

**Table S1. Related Unigenes using nuclear transportation trap (NTT) system and “after suppression subtractive” method**

| **EST ID** | **Highest Homology** | **Genbank accession** | **Organism** | **Copies** | **NLS** |
| --- | --- | --- | --- | --- | --- |
| **Energy and metabolism proteins** | | | | | |
| ScT17 | Phosphoenolpyruvate carboxylase (pepC) gene | AY548429.1 | *Secale cereale* | 1 |  |
| ScT34 | Putative proton pump | AAL78104.1 | *Oryza sativa* | 1 |  |
| ScT42 | DNA binding with one finger 7 protein | AB087852.1 | *Pisum sativum* | 1 | Y |
| ScT55 | Putative chloroplast inner envelope protein | AAG13550.2 | *Oryza sativa* | 1 |  |
| ScT136 | Metal-transporting P-type ATPase-like protein | BAC79908.1 | *Oryza sativa* | 3 | Y |
| ScT137 | ATPase-like protein | BAD31071.1 | *Oryza sativa* | 4 |  |
| ScT178 | Rho-GTPase-activating protein-like | ABE77340.1 | *Hordeum vulgare* | 1 |  |
| ScT372 | Beta-glucosidase | AF293849.1 | *Secale cereale* | 1 |  |
| ScT411 | MTN3 homolog | AAC64192.1 | *Arabidopsis thaliana* | 1 | Y |
| **Resistance-related protein** | | | | | |
| ScT1 | Heat shock protein 90 | JQ685506.1 | *Secale cereale* | 13 | Y |
| ScT3 | CCAAT-binding transcription factor | XM_002893582.1 | *Arabidopsis* | 2 |  |
| ScT4 | Heat-shock protein | Z30243.1 | *Secale cereale* | 2 |  |
| ScT8 | R2R3-MYB Transcription Factor | AK108452.1 | *Oryza sativa* | 3 | Y |
| ScT11 | MIKC-type MADS-box transcription factor WM24B | AM502892.1 | *Triticum aestivum* | 3 | Y |
| ScT13 | Putative transcription factor bHLH | AY222337.1 | *Oryza sativa* | 1 | Y |
| ScT15 | Dehydration responsive element binding protein | AY311483.1 | *Secale cereale* | 3 | Y |
| ScT31 | Hv1LRR2 | AAD46471.1 | *Hordeum vulgare* | 2 |  |
| ScT33 | Putative transcription factor X1 | AAL35831.2 | *Triticum monococcum* | 1 |  |
| ScT35 | Translationally controlled tumor protein | AAM34280.1 | *Triticum aestivum* | 6 | Y |
| ScT36 | Hypersensitive-induced reaction protein 4 | AAN17454.1 | *Hordeum vulgare* | 1 |  |
| ScT37 | Cbf15 gene | HQ730769.1 | *Secale cereale* | 3 | Y |
| ScT38 | Tandem zinc finger protein | AK106392.1 | *Oryza sativa* | 5 | Y |
| ScT39 | Hypersensitive-induced reaction protein 3 | AAN17456.1 | *Hordeum vulgare* | 1 | Y |
| ScT43 | bZIP transcription factor | AB185280.1 | *Oryza sativa* | 2 | Y |
| ScT44 | Transcription factor C2C2-Dof | AB378644.1 | *Lotus japonicus* | 2 | Y |
| ScT48 | Wheat cold induced 16 | AB830333.1 | *Triticum aestivum* | 8 | Y |
| ScT59 | MADS-box transcriptional factor | AAO85374.1 | *Triticum monococcum* | 1 | Y |
| ScT68 | IDS3 | BAB07798.1 | *Hordeum vulgare* | 1 |  |
| ScT78 | Late embryogenesis abundant protein | AY148492.1 | *Triticum aestivum* | 2 | Y |
| ScT89 | ABI5 binding protein B1 | BAG12828.1 | *Triticum aestivum* | 2 | Y |
| ScT90 | AP2/EREBP transcription factor gene | AY319971.1 | *Oryza sativa* | 3 | Y |
| ScT101 | Inducer of CBF Expression 1 | AK102594.1 | *Oryza sativa* | 1 | Y |
| ScT133 | Ethylene-responsive element binding protein 1 | AY781352.1 | *Triticum aestivum* | 12 | Y |
| ScT139 | bZip type transcription factor TaABI5 | BAF36444.1 | *Triticum aestivum* | 2 | Y |
| ScT140 | Putative ethylene-responsive transcription factor | BAG12386.1 | *Hordeum vulgare* | 1 |  |
| ScT141 | ABI5 binding protein A1 | BAG12827.1 | *Triticum aestivum* | 3 | Y |
| ScT143 | ERF transcription factor | CT841997.1 | *Oryza sativa* | 3 | Y |
| ScT144 | C2H2-type zinc finger protein | D16416.1 | *Triticum aestivum* | 4 | Y |
| ScT145 | Translation initiation factor gene | DQ279897.1 | *Secale cereale* | 1 |  |
| ScT157 | MYB transcription factor TaMYB1 | ABC86569.1 | *Triticum aestivum* | 3 | Y |
| ScT161 | Stress-related protein | ABK06393.1 | *Citrus sinensis* | 4 | Y |
| ScT165 | CBFIVa-2A | ABY59786.1 | *Secale cereale* | 2 |  |
| ScT166 | Homeodomain-leucine zipper I-class homeobox protein | ACA29192.1 | *Hordeum vulgare* | 1 |  |
| ScT168 | Cbf2 | ADX32473.1 | *Secale cereale* | 1 |  |
| ScT169 | Cbf6 | ADX32474.1 | *Secale cereale* | 1 | Y |
| ScT171 | Cbf12 | ADX32477.1 | *Secale cereale* | 1 | Y |
| ScT172 | Cbf14 | ADX32478.1 | *Secale cereale* | 2 | Y |
| ScT199 | CBFIIId-12 | ABY59783.1 | *Secale cereale* | 1 | Y |
| ScT205 | Stripe rust-resistance-like protein (RGA) gene | DQ494535.1 | *Secale cereale* | 3 |  |
| ScT206 | MADS-box transcription factor TaAGL36 | DQ512352.1 | *Triticum aestivum* | 1 | Y |
| ScT208 | CBF16 gene | EU076384.1 | *Triticum monococcum* | 2 |  |
| ScT209 | CBFIVa-2B | EU194250.1 | *Secale cereale* | 1 |  |
| ScT222 | Heat shock transcription factor gene | AK066316.1 | *Oryza sativa* | 5 | Y |
| ScT235 | MADS-box protein vrn-1 | EU525891.1 | *Secale cereale* | 1 |  |
| ScT236 | Os01g67210-like protein gene | EU931290.1 | *Secale cereale* | 1 |  |
| ScT238 | Cyclin-like F-box gene | FJ236243.1 | *Secale cereale* | 2 |  |
| ScT254 | Transcription factor MYC4 | EMS55891.1 | *Triticum urartu* | 2 |  |
| ScT269 | Aluminum activated citrate transporter 1-4 | GQ403695.1 | *Secale cereale* | 5 |  |
| ScT270 | Putative zinc finger-CTT domain protein | GU324589.1 | *Secale cereale* | 4 | Y |
| ScT273 | ADH1 (adh1) gene | GU798028.1 | *Oryza sativa* | 2 | Y |
| ScT286 | bZIP transcription factor | FJ194457.1 | *Triticum aestivum* | 4 | Y |
| ScT299 | Hd1-like protein | GU324592.1 | *Secale cereale* | 2 |  |
| ScT301 | CBFIIId-19 | ABY59785.1 | *Secale cereale* | 1 |  |
| ScT308 | Cbf11 | ADX32476.1 | *Secale cereale* | 2 | Y |
| ScT318 | Hypersensitive-induced reaction protein 1 | AAN17457.1 | *Hordeum vulgare* | 1 |  |
| ScT332 | MADS box protein VRT-2 | ADR51708.1 | *Secale cereale* | 2 |  |
| ScT339 | C-myb-like transcription factor | AF190302.1 | *Secale cereale* | 2 | Y |
| ScT340 | Thioredoxin-like protein (Trx) | AF159386.1 | *Secale cereale* | 2 |  |
| ScT365 | MYB3R transcription factor (MYB3R1) | HQ236494.1 | *Triticum aestivum* | 14 | Y |
| ScT367 | Dhn3 | ADX32481.1 | *Secale cereale* | 1 |  |
| ScT369 | Thioredoxin-like protein | AF186240.1 | *Secale cereale* | 1 |  |
| ScT370 | C-myb-like transcription factor (MYB3R-1) | AF190301.1 | *Secale cereale* | 3 | Y |
| ScT373 | CBF-like protein | AF370730.1 | *Secale cereale* | 2 | Y |
| ScT374 | Early-methionine-labelled polypeptide em-R1 gene | AJ011951.1 | *Secale cereale* | 2 | Y |
| ScT375 | Erebp gene for ethylene response element binding protein | AJ515477.2 | *Triticum aestivum* | 6 | Y |
| ScT376 | Ocs-element binding factor 1 (obf1 gene) | AJ617794.1 | *Secale cereale* | 1 |  |
| ScT378 | Heat and cold induced 1 | AK067013.1 | *Oryza sativa* | 2 | Y |
| ScT380 | Ethylene-responsive transcriptional factor | AK069262.1 | *Oryza sativa* | 4 | Y |
| ScT382 | Metal tolerance protein | AK100735.1 | *Oryza sativa* | 2 |  |
| ScT384 | ABRE-binding protein responding to ABA and glucose | AK103188.1 | *Oryza sativa* | 3 | Y |
| ScT387 | AMP-binding protein gene | AK106615.1 | *Oryza sativa* | 1 |  |
| ScT389 | Late embryogenesis abundant protein gene | AK107973.1 | *Oryza sativa* | 3 | Y |
| ScT401 | Cold regulated LTCOR18 | AAC02689.1 | *Lavatera thuringiaca* | 8 | Y |
| ScT405 | bZIP transcription factor, partial | AAO06905.1 | *Hordeum vulgare* | 3 | Y |
| **Transporters** | | | | | |
| ScT7 | Acyl carrier protein III | AK108342.1 | *Oryza sativa* | 3 |  |
| ScT14 | Phosphate transporter 2-1 | AY293827.1 | *Triticum aestivum* | 1 |  |
| ScT18 | 14-3-3 protein | AY736127.1 | *Triticum aestivum* | 4 | Y |
| ScT41 | mRNA transport factor | AAY84882.1 | *Triticum aestivum* | 3 | Y |
| ScT47 | ATP-dependent Clp protease proteolytic subunit | AB746947.1 | *Secale cereale* | 1 |  |
| ScT80 | Half-size adenosine triphosphate-binding cassette transporter subgroup G | AK120079.1 | *Oryza sativa* | 2 |  |
| ScT88 | Glucosyltransferase | AB548283.1 | *Secale cereale* | 2 |  |
| ScT91 | Putative citrate efflux MATE transporter | AB571882.1 | *Secale cereale* | 3 |  |
| ScT135 | Protein kinase | BAB61736.1 | *Hordeum vulgare* | 1 |  |
| ScT160 | GSK-like kinase 1A | ABG29422.1 | *Triticum aestivum* | 1 |  |
| ScT162 | Glutathione S-transferase 1 | ABK41475.1 | *Triticum monococcum* | 2 |  |
| ScT379 | RING finger E3 ligase | AK067013.1 | *Oryza sativa* | 2 |  |
| ScT381 | Histone Deacetylase | AK072845.1 | *Oryza sativa* | 1 | Y |
| ScT388 | ABC Transporter Protein | AK106792.1 | *Oryza sativa* | 6 |  |
| **Signaling proteins** | | | | | |
| ScT2 | Tetratricopeptide repeat containing protein | JX424308.1 | *Triticum monococcum* | 3 |  |
| ScT10 | Predicted protein | AK353965.1 | *Hordeum vulgare* | 1 |  |
| ScT156 | Serine/threonine kinase gene | ABC73058.1 | *Hordeum vulgare* | 2 |  |
| ScT159 | Serine/threonine kinase-like protein ABC1040 | ABF18540.1 | *Hordeum vulgare* | 3 |  |
| ScT173 | Putative 9-cis-epoxycarotenoid dioxygenase | BAF02837.1 | *Hordeum vulgare* | 1 |  |
| ScT239 | Protein kinase gene | FJ236251.1 | *Secale cereale* | 1 |  |
| ScT272 | Puma pseudo-response regulator (PRR) gene | GU324593.1 | *Secale cereale* | 5 | Y |
| ScT385 | RING-C2 type protein | AK103750.1 | *Oryza sativa* | 2 | Y |
| **Unknown proteins** | | | | | |
| ScT5 | Unknown protein |  |  | 1 | Y |
| ScT57 | Unknown protein |  |  | 1 |  |
| ScT62 | Unknown protein |  |  | 1 | Y |
| ScT64 | Unknown protein |  |  | 1 |  |
| ScT65 | Unknown protein |  |  | 1 | Y |
| ScT66 | Unknown protein |  |  | 1 |  |
| ScT67 | Unknown protein |  |  | 1 |  |
| ScT69 | Unknown protein |  |  | 1 |  |
| ScT70 | Unknown protein |  |  | 1 | Y |
| ScT71 | Unknown protein |  |  | 1 |  |
| ScT81 | Unknown protein |  |  | 1 |  |
| ScT83 | Unknown protein |  |  | 1 |  |
| ScT86 | Unknown protein |  |  | 1 |  |
| ScT87 | Unknown protein |  |  | 1 |  |
| ScT92 | Unknown protein |  |  | 1 | Y |
| ScT95 | Unknown protein |  |  | 1 |  |
| ScT98 | Unknown protein |  |  | 1 |  |
| ScT99 | Unknown protein |  |  | 1 |  |
| ScT102 | Unknown protein |  |  | 1 |  |
| ScT103 | Unknown protein |  |  | 1 |  |
| ScT104 | Unknown protein |  |  | 1 |  |
| ScT105 | Unknown protein |  |  | 1 | Y |
| ScT106 | Unknown protein |  |  | 1 |  |
| ScT107 | Unknown protein |  |  | 1 |  |
| ScT108 | Unknown protein |  |  | 1 |  |
| ScT109 | Unknown protein |  |  | 1 |  |
| ScT111 | Unknown protein |  |  | 1 | Y |
| ScT114 | Unknown protein |  |  | 1 |  |
| ScT117 | Unknown protein |  |  | 1 |  |
| ScT142 | Unknown protein |  |  | 2 |  |
| ScT147 | Unknown protein |  |  | 4 |  |
| ScT148 | Unknown protein |  |  | 1 |  |
| ScT149 | Unknown protein |  |  | 1 |  |
| ScT151 | Unknown protein |  |  | 1 |  |
| ScT152 | Unknown protein |  |  | 1 |  |
| ScT153 | Unknown protein |  |  | 1 | Y |
| ScT154 | Unknown protein |  |  | 1 |  |
| ScT158 | Unknown protein |  |  | 1 |  |
| ScT164 | Unknown protein |  |  | 1 |  |
| ScT196 | Unknown protein |  |  | 11 | Y |
| ScT210 | Unknown protein |  |  | 1 |  |
| ScT215 | Unknown protein |  |  | 1 |  |
| ScT223 | Unknown protein |  |  | 1 |  |
| ScT225 | Unknown protein |  |  | 1 |  |
| ScT227 | Unknown protein |  |  | 1 |  |
| ScT237 | Unknown protein |  |  | 1 |  |
| ScT240 | Unknown protein |  |  | 3 | Y |
| ScT241 | Unknown protein |  |  | 1 |  |
| ScT242 | Unknown protein |  |  | 1 | Y |
| ScT244 | Unknown protein |  |  | 1 |  |
| ScT245 | Unknown protein |  |  | 1 | Y |
| ScT246 | Unknown protein |  |  | 1 |  |
| ScT247 | Unknown protein |  |  | 1 | Y |
| ScT275 | Unknown protein |  |  | 3 | Y |
| ScT355 | Unknown protein |  |  | 1 |  |
| ScT358 | Unknown protein |  |  | 1 |  |
| ScT364 | Unknown protein |  |  | 1 | Y |
| ScT368 | Unknown protein |  |  | 1 |  |
| ScT371 | Unknown protein |  |  | 1 |  |
| ScT386 | Unknown protein |  |  | 1 |  |
| ScT406 | Unknown protein |  |  | 2 | Y |
| ScT407 | Unknown protein |  |  | 1 |  |
| ScT485 | Unknown protein |  |  | 1 | Y |

**Table S2. Remove Unigenes using “after suppression subtractive” method from two libraries**

| **EST ID** | **Highest Homology** | **Genbank accession** | **Organism** | **Copies** | **NLS** |
| --- | --- | --- | --- | --- | --- |
| ScT75 | 75k gamma secalin | AFX60464.1 | *Secale cereale* | 1 |  |
| ScT390 | actin | FJ032189.1 | *Secale cereale* | 6 |  |
| ScT6 | auxin efflux carrier | AK063976.1 | *Oryza sativa* | 1 |  |
| ScT12 | auxin repressed protein (ARP) | KF155508.1 | *Oryza sativa* | 2 |  |
| ScT113 | beta-tubulin | FJ032190.1 | *Secale cereale* | 6 |  |
| ScT110 | BX6-like gene | HG380520.1 | *Secale cereale* | 1 |  |
| ScT121 | catalase (cat1a gene) | Z54143.2 | *Secale cereale* | 1 |  |
| ScT112 | cellulose synthase-like protein H1 | ACN67534.1 | *Hordeum vulgare* | 1 |  |
| ScT132 | chalcone isomerase | AAM13449.1 | *Hordeum vulgare* | 2 |  |
| ScT129 | chalcone-flavanone isomerase (Chi-R1) gene | KC788195.1 | *Secale cereale* | 4 |  |
| ScT115 | chloroplast indole-3-glycerol phosphate lyase | JQ716987.1 | *Secale cereale* | 2 |  |
| ScT116 | DNA methyltransferase | AK065147.1 | *Oryza sativa* | 1 |  |
| ScT127 | F-box LRR-repeat protein 2 gene | FJ374551.1 | *Secale cereale* | 1 |  |
| ScT118 | F-box protein | AK121543.1 | *Oryza sativa* | 2 |  |
| ScT119 | ferritin 1A | ACJ05646.1 | *Triticum aestivum* | 2 |  |
| ScT120 | fructose-bisphosphate aldolase gene | FJ403590.1 | *Secale cereale* | 1 |  |
| ScT179 | Glossy1-homologous gene | AK102938.1 | *Oryza sativa* | 1 |  |
| ScT180 | IAA biosynthesis gene | AK071687.1 | *Oryza sativa* | 1 | Y |
| ScT181 | kinase R-like protein | AAL51071.1 | *Triticum aestivum* | 1 |  |
| ScT182 | Magnesium Transporter Gene | AB731703.1 | *Oryza sativa* | 1 |  |
| ScT183 | phytoene synthase 1 | ACF72684.1 | *Triticum monococcum* | 1 |  |
| ScT184 | protochlorophyllide oxidoreductase A | AK065236.1 | *Oryza sativa* | 1 |  |
| ScT185 | ribosomal protein S7 | AF118149.1 | *Secale cereale* | 2 |  |
| ScT186 | ROGO-36 omega secalin gene | FJ561451.1 | *Secale cereale* | 1 |  |
| ScT187 | storage protein | X02602.1 | *Secale cereale* | 2 |  |
| ScT403 | thaumatin-like protein 1 precursor (TLP1) | AF096927.1 | *Secale cereale* | 1 | Y |
| ScT391 | VIL3 protein | ABJ99749.1 | *Triticum monococcum* | 2 | Y |
| ScT392 | VRN1 | AAO85376.2 | *Triticum monococcum* | 3 |  |
| ScT393 | VRN-3 | ADR51710.1 | *Secale cereale* | 4 |  |
| ScT394 | VRN-D1 | AAW73218.1 | *Triticum aestivum* | 3 |  |
| ScT395 | waxy | ACM41699.1 | *Secale cereale* | 5 |  |
| ScT9 | unknown protein |  |  | 1 |  |
| ScT16 | unknown protein |  |  | 1 |  |
| ScT22 | unknown protein |  |  | 1 |  |
| ScT23 | unknown protein |  |  | 1 |  |
| ScT25 | unknown protein |  |  | 1 |  |
| ScT26 | unknown protein |  |  | 1 |  |
| ScT28 | unknown protein |  |  | 1 |  |
| ScT29 | unknown protein |  |  | 1 | Y |
| ScT40 | unknown protein |  |  | 1 |  |
| ScT45 | unknown protein |  |  | 1 |  |
| ScT52 | unknown protein |  |  | 1 |  |
| ScT53 | unknown protein |  |  | 1 | Y |
| ScT73 | unknown protein |  |  | 1 |  |
| ScT74 | unknown protein |  |  | 1 |  |
| ScT76 | unknown protein |  |  | 1 |  |
| ScT77 | unknown protein |  |  | 1 |  |
| ScT190 | unknown protein |  |  | 1 |  |
| ScT191 | unknown protein |  |  | 1 |  |
| ScT192 | unknown protein |  |  | 1 |  |
| ScT193 | unknown protein |  |  | 1 |  |
| ScT201 | unknown protein |  |  | 1 | Y |
| ScT202 | unknown protein |  |  | 1 |  |
| ScT224 | unknown protein |  |  | 1 |  |
| ScT232 | unknown protein |  |  | 1 |  |
| ScT238 | unknown protein |  |  | 1 |  |
| ScT248 | unknown protein |  |  | 1 |  |
| ScT249 | unknown protein |  |  | 1 |  |
| ScT258 | unknown protein |  |  | 1 |  |
| ScT298 | unknown protein |  |  | 1 |  |
| ScT305 | unknown protein |  |  | 1 |  |
| ScT344 | unknown protein |  |  | 1 |  |
| ScT345 | unknown protein |  |  | 1 |  |
| ScT347 | unknown protein |  |  | 1 |  |
| ScT348 | unknown protein |  |  | 1 |  |
| ScT350 | unknown protein |  |  | 1 |  |
| ScT351 | unknown protein |  |  | 1 |  |
| ScT352 | unknown protein |  |  | 1 | Y |
| ScT353 | unknown protein |  |  | 1 |  |
| ScT356 | unknown protein |  |  | 1 |  |
| ScT377 | unknown protein |  |  | 1 |  |
| ScT398 | unknown protein |  |  | 1 | Y |

**Table S 3. PCR primers for Q-RT-PCR analysis of gene expression**

| **Gene** | **Forward primer(5’-3’)** | | **Reverse primer (5′-3′)** | |
| --- | --- | --- | --- | --- |
| *SaT1* | GACCGAGACCTTCGCCTTCC | | TGGTCATGCCAATGCCGCTGTC | |
| *SaT36* | TGGTGAACCGAACACTGACA | | AGAACCACAAGTGCCCAATC | |
| *SaT133* | TGGGAGAATGATATCAAGACCCCTG | | CAAGGGCATGTCATCAAAGGTC | |
| *SaT196* | GGCAGCGCCTGCGCCTGCCCCTGCC | | CAGAAGTAACGCAGCGCACAAAC | |
| *Actin* | TACTCCCTCACAACAACC | | GCTCCTGCTCATAATCAA | |
|  | |  | |  |
