# Supplementary figures and images for: An iNTT system for the large-scale screening of differentially expressed, nuclear-targeted proteins: cold-treatment-induced nucleoproteins in Rye (Secale cereale L.)
Source: BMC Genomics. 2016 Mar 5;17:189. doi: 10.1186/s12864-016-2548-y (PMC4779243; doi:10.1186/s12864-016-2548-y)

**Figure S1. Experimental protocol of iNTT system method**

###
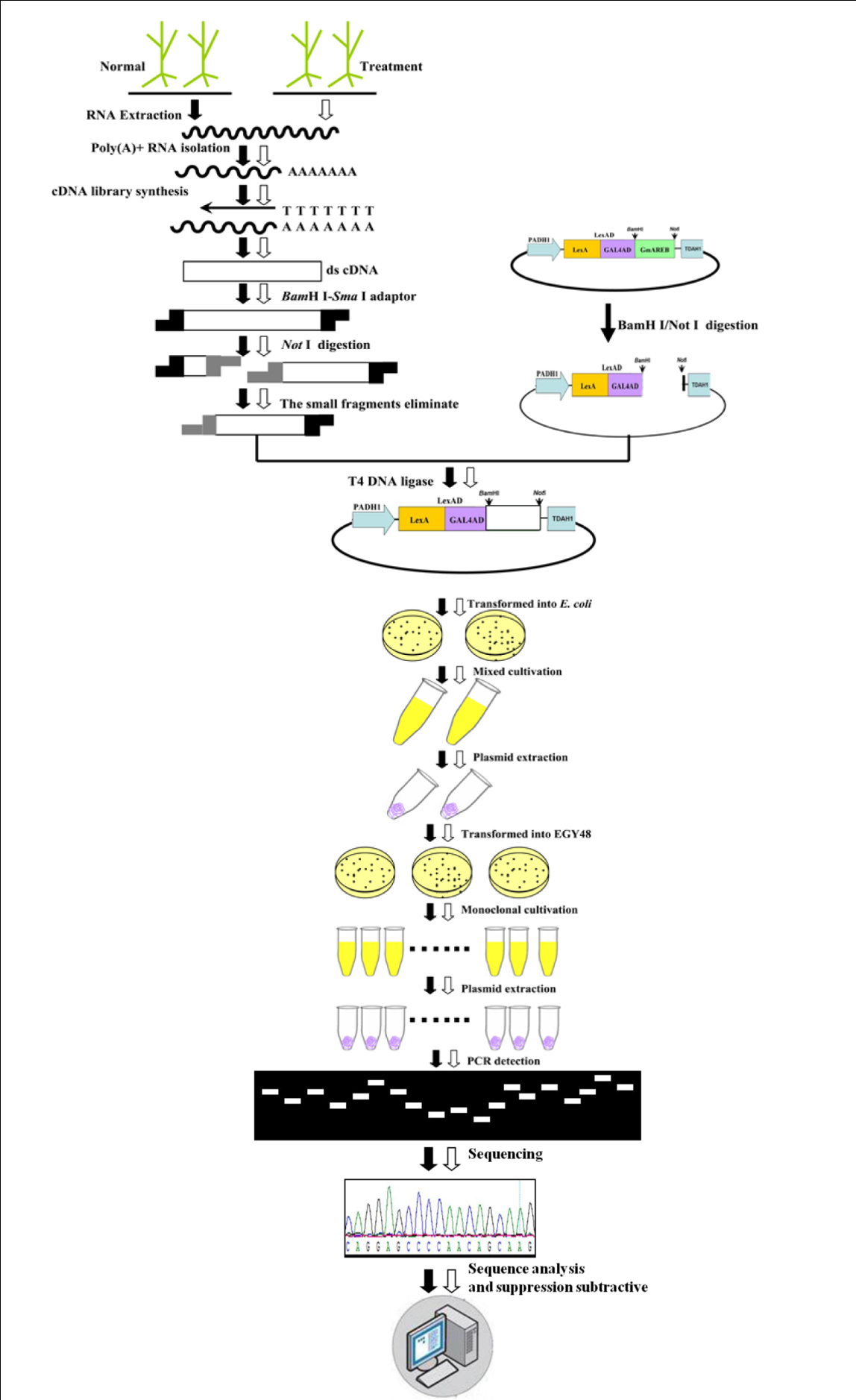

Supplement: Additional file 2: Figure S1. — Experimental protocol of iNTT system method. (DOCX 538 kb) [file 12864_2016_2548_MOESM2_ESM.docx]
